# Photoacid behaviour in a fluorinated green fluorescent protein chromophore: ultrafast formation of anion and zwitterion states†
†Electronic supplementary information (ESI) available: Description of the fluorescence up-conversion experiment, additional information supporting evidence of ESPT, details of the spectral decomposition and notes on synthesis and characterization of DF*p*-HBDI. See DOI: 10.1039/c6sc02031c


**DOI:** 10.1039/c6sc02031c

**Published:** 2016-06-06

**Authors:** S. P. Laptenok, J. Conyard, P. C. Bulman Page, Y. Chan, M. You, S. R. Jaffrey, S. R. Meech

**Affiliations:** a School of Chemistry , University of East Anglia , Norwich NR4 7TJ , UK . Email: S.Meech@uea.ac.uk; b Department of Pharmacology Weill Medical College , Cornell University , 1300 York Avenue, Box 70 , New York , NY 10065 , USA

## Abstract

The photophysics of the chromophore of the green fluorescent protein in *Aequorea victoria* (avGFP) are dominated by an excited state proton transfer reaction. In contrast the photophysics of the same chromophore in solution are dominated by radiationless decay, and photoacid behaviour is not observed. Here we show that modification of the p*K*_a_ of the chromophore by fluorination leads to an excited state proton transfer on an extremely fast (50 fs) time scale. Such a fast rate suggests a barrierless proton transfer and the existence of a pre-formed acceptor site in the aqueous solution, which is supported by solvent and deuterium isotope effects. In addition, at lower pH, photochemical formation of the elusive zwitterion of the GFP chromophore is observed by means of an equally fast excited state proton transfer from the cation. The significance of these results for understanding and modifying the properties of fluorescent proteins are discussed.

## Abstract 2

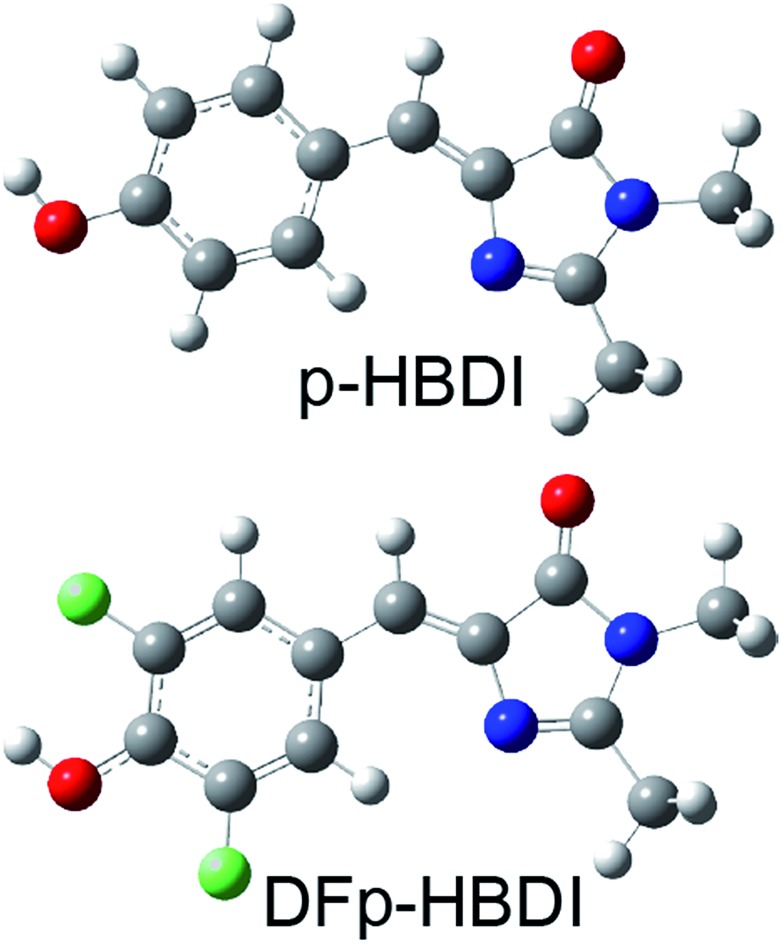
The photophysics of the chromophore of the green fluorescent protein in *Aequorea victoria* (avGFP) are dominated by an excited state proton transfer reaction.

## Introduction

**Fig. 1:**
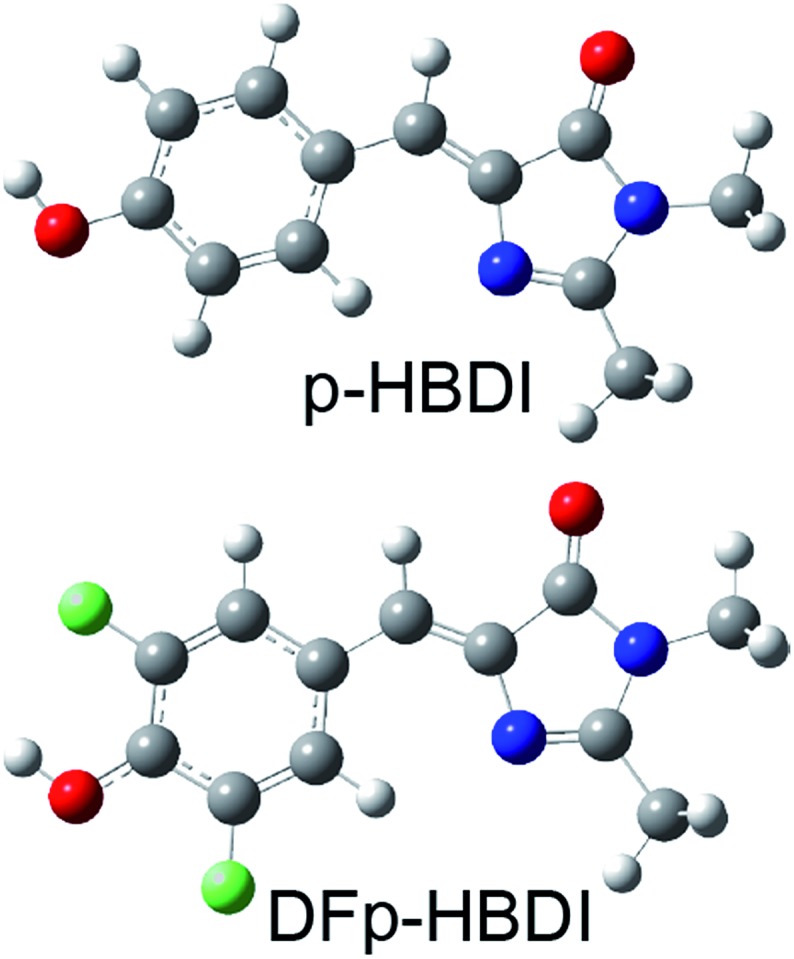
Structures of *p*-HBDI and DF*p*-HBDI.

The green fluorescent protein isolated from *Aequorea victoria* (avGFP) is the founding member of the fluorescent protein (FP) family which underpins some of the most important developments in bioimaging of the past two decades.^1^–^3^ The chromophore of avGFP, which is common to most FPs, is formed in a post-translational intramolecular cyclisation and oxidation reaction.^4^ The chromophore exists in nature in both neutral and anionic forms, and in avGFP these were shown to be coupled by an ultrafast excited state proton transfer (ESPT) reaction, with the proton acceptor being a glutamic acid residue (E222) connected to the phenolic hydroxyl group of the chromophore by a three step proton wire.^5^–^7^ The complete proton transfer cycle in avGFP has been characterized in considerable detail in both ground and excited states, and these studies have provided new insights into proton transfer along proton wires.^7^–^10^ In sharp contrast to the dominant photoacid properties of the chromophore in avGFP, the photophysics of its synthetic analogue (*p*-hydroxybenzylideneimidazolinone, *p*-HBDI, Fig. 1) in solution are dominated by ultrafast radiationless decay, and no ESPT has been observed in any solvent.^11^,^12^ This behaviour is repeated in a number of synthetic analogues of *p*-HBDI.^13^ Indeed Solntsev and co-workers showed that ESPT in *p*-HBDI could only be induced by locking the chromophore in a three ring structure. By thus extending the excited state lifetime, ESPT was observed to occur two to three orders of magnitude slower than in the protein.^14^ Solntsev *et al.* also showed that the meta hydroxy derivative, *m*-HBDI, does exhibit picosecond ESPT in aqueous solution,^15^ while Hsieh *et al.* showed that *o*-HBDI in aprotic solvents undergoes efficient intramolecular ESPT, protonating the imidazole nitrogen with which the *o*-OH group forms an intramolecular H-bond.^16^ However, neither the *o*-HBDI nor the *m*-HBDI isomers of the chromophore occur naturally in the FP family. In this work we show that modification of chromophore p*K*_a_ by fluorination leads to exceptionally fast (50 fs) photoacid behavior for both the neutral and cation forms, with the latter case revealing emission of the elusive zwitterion.

## Results and discussion

**Fig. 2:**
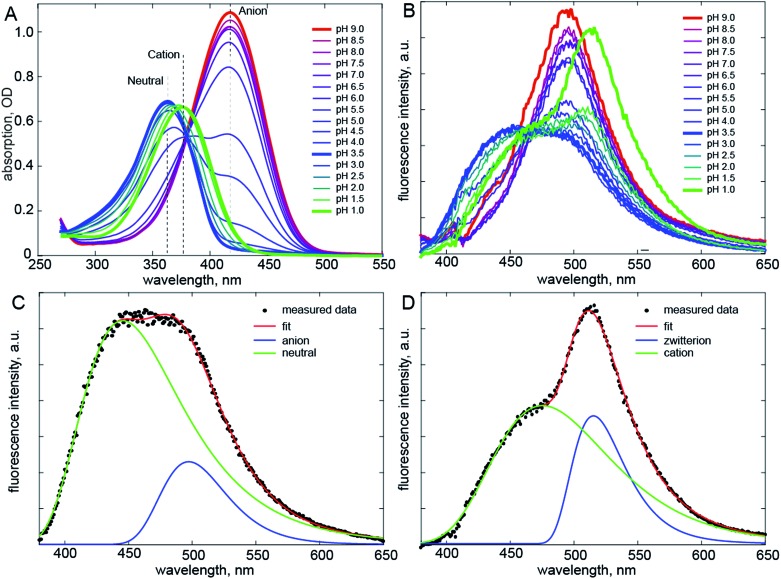
Electronic spectra. (A) Absorption spectra of DF*p*-HBDI measured as a function of pH. (B) pH Dependent emission spectra. Shown in bold are emission of the anion (red, pH 9), neutral (blue, pH 3.5) and cation (green, pH 1) forms of DF*p*-HBDI; the latter two are clearly bimodal. (C) The spectral decomposition (see also Fig. S2†) for the neutral form, where the anion spectrum was obtained from fitting the pH 9 data and only its amplitude was fit. (D) Similar decomposition for emission following cation excitation. The excitation wavelength was 360 nm.

Recently a range of new derivatives of *p*-HBDI were synthesized with a view to developing fluorescent RNA aptamers for RNA based imaging. Highly fluorescent aptamers binding specific FP chromophores were generated by a systematic evolution approach.^17^–^19^ One of the most successful incorporated difluoro-*p*-HBDI (DF*p*-HBDI, Fig. 1) as the chromophore, yielding the ‘Spinach’ RNA^17^ (which was recently supplanted by the more thermally stable ‘Spinach 2’ aptamer^20^). Details of the synthesis and characterization of DF*p*-HBDI are presented in ESI.† The electronic spectra of DF*p*-HBDI in aqueous solution as a function of pH are shown in Fig. 2. The absorption spectra (Fig. 2A) are uniformly blue shifted relative to those of *p*-HBDI itself (Table 1, Fig. S1†). At higher pH the anionic (phenolic deprotonated) form is observed, while at reduced pH the neutral form is seen. The p*K*_a_ for the anion to neutral conversion of DF*p*-HBDI is 5.4, a decrease of 2.4 p*K*_a_ units from *p*-HBDI. Further reduction in the pH results in formation of the cation (usually assumed to be N-protonated at the imidazole ring, Fig. S1†) with a p*K*_a_ of 1.5 for DF*p*-HBDI, compared to 2.7 for *p*-HBDI. The cation is red-shifted with respect to the neutral form for both derivatives. Peak wavelengths are reported in Table 1. Thus, the main effect of meta substitution of the electron withdrawing fluorine atoms on the absorption spectra is a blue-shift and a significant reduction in the anion/neutral/cation p*K*_a_ values (Table 1). The rather small spectral shift on fluorination is noteworthy, when compared to the very large red shift seen for substitution with weak electron donors (*e.g. m*-dimethyl *p*-HBDI).^13^ In contrast to their qualitatively similar absorption spectra, the pH dependent emission spectra of DF*p*-HBDI (Fig. 2B) are very different to those of *p*-HBDI (shown in Fig. S1†), although the emission remains very weak in both cases (fluorescence quantum yield < 10^–4^). For *p*-HBDI the emission spectrum is always a single band (Table 1, Fig. S1†). On excitation of the DF*p*-HBDI anion (pH > 7) a single emission spectrum is also observed with a maximum at 500 nm, which is similar to but slightly red-shifted compared to HBDI, with the result that the Stokes shift is increased on fluorination; similarly the fluorescence of neutral DF*p*-HBDI in methanol is also a single band (Fig. S4A†). However, excitation of the neutral form of DF*p*-HBDI (pH 3.5) near its peak wavelength in aqueous solution leads to an emission spectrum which is clearly bimodal, with a broad contribution at 445 nm assigned to the neutral emission and another at 500 nm, characteristic of the anionic form (Fig. 2B). This was confirmed through spectral decomposition into two components, where the anion component was fixed at the profile of the pH 9 emission spectrum excited at 400 nm (see ESI†). This fit gave an accurate description of the whole spectrum (Fig. 2C). The neutral emission spectrum thus recovered is broad, a feature also observed in avGFP.^21^ This observation of anionic emission on neutral excitation in aqueous solution is strongly suggestive of ESPT in DF*p*-HBDI from solute to solvent.

**Table 1:** pH Dependent peak maxima for DF*p*-HBDI compared with the parent compound *p*-HBDI^*a*^

		*p*-HBDI	DF*p*-HBDI
*λ*_max_ absorption/nm	Anion	425	418
	Neutral	370	363
	Cation	391	377
*λ*_max_ emission/nm	Anion	494	500
	Neutral	448	445/500
	Cation	465	475/515
p*K*_a_	Neutral	7.8	5.4
	Cation	2.7	1.5

^*a*^Emission maxima were obtained from spectral decomposition (See ESI).

Surprisingly, when the pH is reduced further and the cation is excited at 360 nm its emission is dominated by a band at 515 nm markedly to the red of even the anion emission, with a second band (a shoulder) resolved at *ca.* 475 nm (Fig. 2D). Again spectral decomposition requires only two components (Fig. S2B,† Table 1). The blue shifted shoulder is assigned to the normal cation emission and the red shifted band to a zwitterionic product of ESPT from the cation to the solvent. The zwitterion is not observed in *p*-HBDI, and has proven elusive in any form of FP chromophore.^22^ It was proposed some time ago that the chromophore in avGFP might be the zwitterion, as its absorption and emission spectra are markedly red shifted relative to the chromophore in solution.^23^ However, no further evidence has been found to support this proposal. The present results confirm that the zwitterion emission is indeed strongly red-shifted (for DF*p*-HBDI) but also that no stable ground state form was observed. Thus, the present and earlier data suggest that for *p*-HBDI itself the zwitterion has not been observed, and that the emission of avGFP arises from the neutral and anionic forms.

**Fig. 3:**
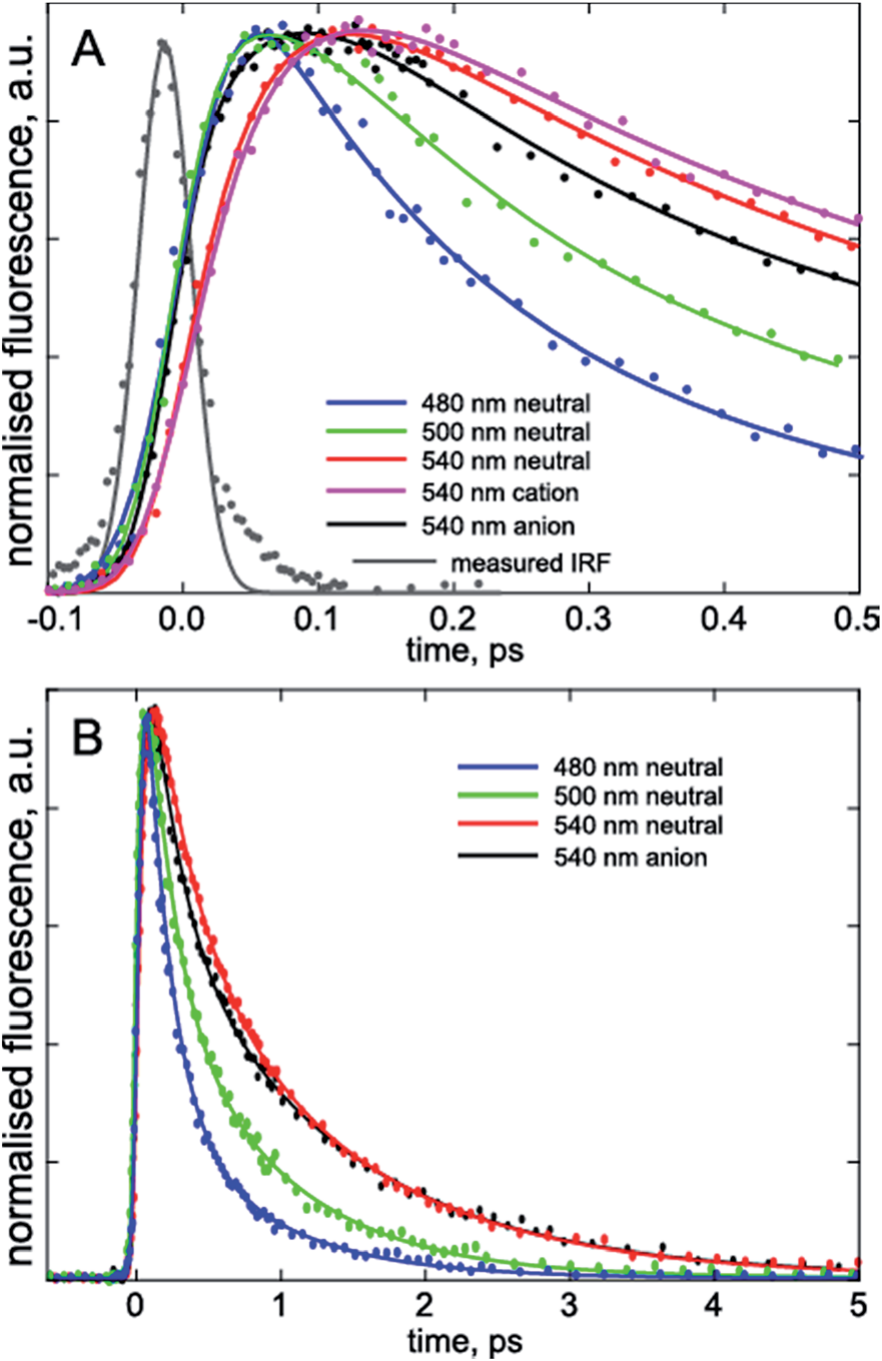
Wavelength resolved time resolved fluorescence. The three charge forms studied are shown on the sub-picosecond (A) and picosecond (B) time scales. In (A) the grey curve is the instrument response function used in the convolution analysis; note the clear rise seen at 540 nm for the neutral but absent for the anion measured at the same wavelength, indicating the requirement for a fitted risetime (Table 2).

**Fig. 4:**
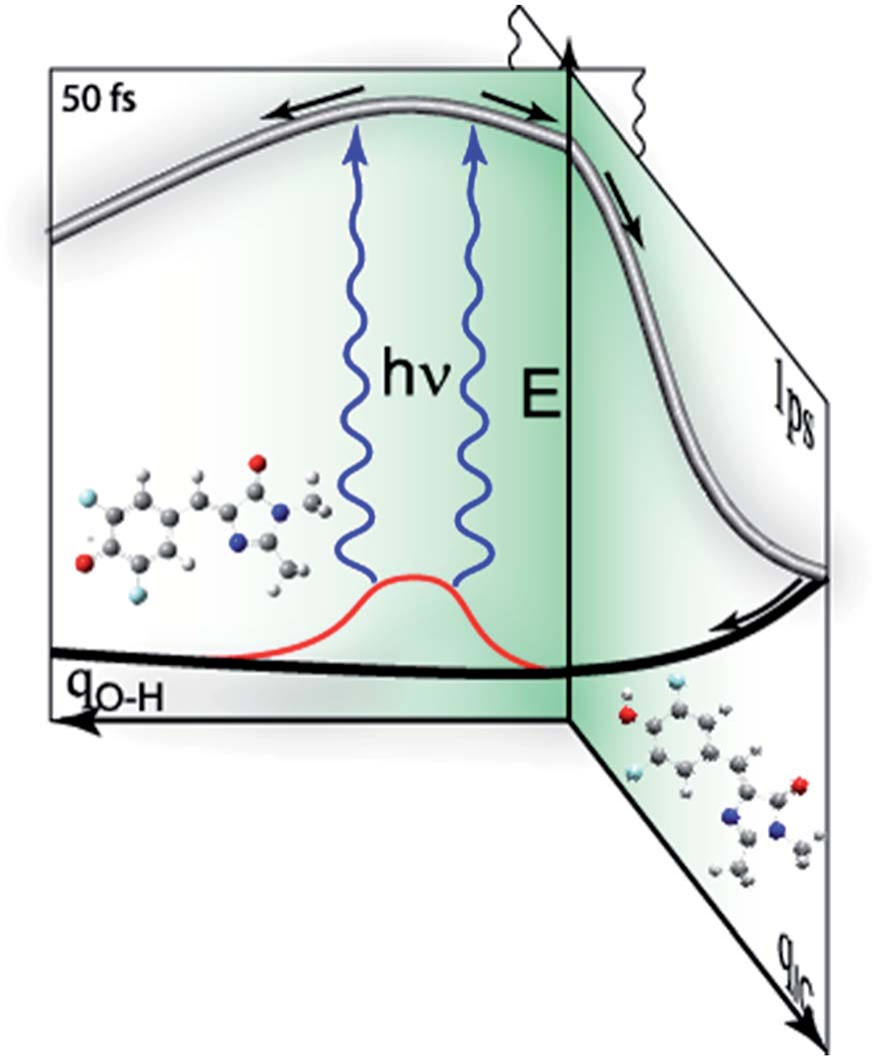
Schematic representation of competing decay pathways in DF*p*-HBDI. The neutral form of the chromophore can exist in a range of environments (red) which either do or do not lead to ESPT on electronic excitation. Thus electronic excitation may lead directly to ESPT, to a pre-formed trap state reached along the proton transfer coordinate, *q*_OH_, in 50 fs. Alternatively, the neutral form lacking an acceptor state undergoes the normal *ca.* 1 ps decay characteristic of other HBDI analogs, along a coordinate leading to internal conversion, *q*_IC_.

To confirm that the bimodal emissions observed in Fig. 2 are due to ESPT rather than, for example, a mixture of ground states with very different fluorescence quantum yields, time resolved measurements are essential. The most direct probe of excited state dynamics in general and ESPT in particular is time resolved fluorescence. It is already established that *p*-HBDI exhibits ultrafast internal conversion^24^,^25^ so sub-picosecond fluorescence resolution will be required to resolve the ESPT reaction. In recent years the fluorescence up-conversion method (see ESI†) has been improved to yield sub-50 fs time resolution.^26^,^27^ This method was applied to DF*p*-HBDI (Fig. 3, Table 2), where the excitation wavelength was 400 nm (the fluorescence spectra measured with 400 nm excitation are very similar to the 360 nm data of Fig. 2, as described in ESI, Fig. S5†). The fluorescence decay of the DF*p*-HBDI anion is nearly independent of wavelength, although slightly shorter on the blue edge, probably due to emission from vibrationally hot states (see Fig. S2† and associated description). This anion decay is however non single exponential (as also observed for *p*-HBDI^24^) with a mean decay time of 0.97 ps. The neutral form of DF*p*-HBDI in methanol also shows a wavelength independent, approximately exponential, decay (Fig. S4B†). In contrast the decay of neutral DF*p*-HBDI in aqueous solution is strongly wavelength dependent with a dominant decay component of <200 fs at 480 nm, where the neutral emission makes the major contribution (Fig. 3 and Table 2). There is also a significant contribution from a longer component of 1.0 ± 0.2 ps at all wavelengths. Importantly, on the red side of the emission, where the anion fluorescence dominates (Fig. 2) a 50 fs risetime is observed. This 50 fs rise is consistent with ultrafast ESPT to form the anion. Although this represents an exceptionally fast risetime, it is readily resolved in the ultrafast up conversion experiment (Fig. 3A). When the same experiment was repeated at pH 1.0, where the DF*p*-HBDI cation dominates the absorption at 400 nm, the same result was recovered, a sub 200 fs decay on the blue edge and a 50 fs risetime in the zwitterion emission (Table 2, Fig. S6†); again, the risetime is indicative of an ultrafast ESPT reaction. It is significant that the neutral decay at 480 nm is bimodal with sub 200 fs and 1 ± 0.2 ps components. The longer component we ascribe to emission from a population of neutral chromophores which do not undergo ESPT but relax *via* fast internal conversion. The observation of emission from both quenched (by ESPT) and unquenched populations of neutral (and cation) forms suggests that the proton acceptor sites for the ESPT reactions are pre-formed in the ground state. Those chromophores without pre-formed acceptor sites undergo ultrafast internal conversion on the longer (*ca.* 1 ps) timescale typical of *p*-HBDI decay instead of ESPT. This two-coordinate decay pathway is illustrated in Fig. 4.

**Table 2:** Fluorescence decay data^*a*^

	nm	*α*	*τ*_1_ ps	*τ*_2_ ps	〈*τ*〉 ps ps	Rise/ps
**DF*p*-HBDI**						
Anion	480	0.60	0.3 ± 0.05	1.4 ± 0.2	0.74	
	500	0.53	0.4 ± 0.1	1.6 ± 0.4	0.96	
	540	0.60	0.5 ± 0.1	1.7 ± 0.4	0.98	
Neutral	480	0.75	0.18 ± 0.1	0.8 ± 0.3	0.34	
	500	0.60	0.18 ± 0.1	0.8 ± 0.1	0.43	0.05 ± 0.1
	540	0.50	0.5 ± 0.1	1.2 ± 0.2	0.85	0.05 ± 0.1
Cation	480	0.75	0.17 ± 0.07	0.7 ± 0.2	0.30	
	500	0.65	0.18 ± 0.08	0.8 ± 0.1	0.40	0.05 ± 0.02
	540	0.34	0.25 ± 0.2	1.1 ± 0.1	0.81	0.05 ± 0.02

***p*-HBDI**						
Anion	500	0.48	0.26 ± 0.05	1.2 ± 0.1	0.74	
Neutral	500	0.74	0.19 ± 0.03	0.56 ± 0.1	0.29	

^*a*^Data were fit to two exponentially decaying components (weights *α* and 1 – *α*) plus a rise when required. Fig. S7 illustrates the need for a rising component at 500 nm, which is not immediately apparent in Fig. 3.

The fact that the 50 fs rise on the red edge due to ESPT is faster than the sub 200 fs blue edge decay for both neutral and cation forms (Table 2) deserves comment, as this suggests an apparent departure from simple two-state kinetics. We ascribe the longer (but sub 200 fs) 480 nm decay to a mixture of the expected ultrafast 50 fs ESPT component with a slower but non-single exponential decay of the neutral DF*p*-HBDI population, which does not undergo ESPT (Fig. 4). The analysis of this sum of a 50 fs decaying population with a longer lived non-single exponentially decaying population in terms of a sum of only two exponentials will return a fast component longer than 50 fs, as observed (Table 2). Because of these multiple contributions to the 480 nm emission, the risetime of the anion (or zwitterion) is a better indication of the rate of ESPT.

All three charge forms of DF*p*-HBDI were also studied in D_2_O, and no kinetic isotope effect was observed (Table S1†). Huppert and co-workers made detailed studies of very efficient intramolecular ESPT reactions, typically occurring on the timescale of 100 fs or longer.^28^,^29^ They found that in derivatives where ESPT became faster, corresponding to a lower barrier along the proton transfer coordinate, the kinetic isotope effect decreased. The negligible kinetic isotope effect seen here thus suggests a barrierless pathway for the ESPT reaction in DF*p*-HBDI in aqueous solution, which is in-turn consistent with the observed 50 fs proton transfer time.

Such an ultrafast proton transfer suggests extreme photoacid behaviour. We can investigate this more quantitatively by using a Forster cycle to calculate the p*K*_a_ of the excited electronic state of DF*p*-HBDI, 
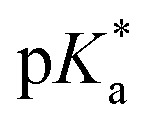
.^30^,^31^ This is obtained spectroscopically from:
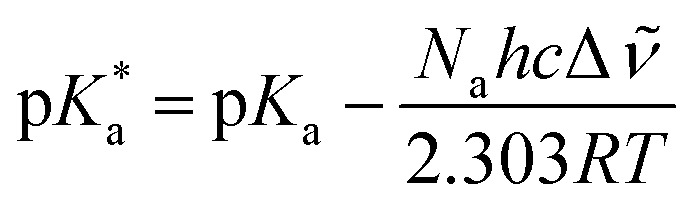


In which Δ*ν̃* is the spectral shift between neutral and anion absorption, which is estimated from the peak maxima as 4500 cm^–1^. The calculation leads to a 
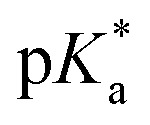
 of –4, which, although less negative than those reported by Simkovich *et al.*,^30^ places DF*p*-HBDI firmly in the class of super-photacids (
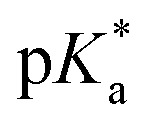
 < –2). However, the rate of ESPT seems to be not only a function of the solute (donor), but also of the solvent (acceptor). We have found that only a fraction of the excited states undergo ESPT (Fig. 4) suggesting the need for a preformed acceptor site; the nature of this site is unclear, but we speculate that it may involve ordering in the solvent proton acceptor, possibly induced by the fluorine atoms, giving them two roles in the ultrafast proton transfer (reduced p*K*_a_ and acceptor structure). The remaining DF*p*-HBDI population decays by internal conversion on the subpicosecond time scale. This conclusion raises the possibility that DF*p*-HBDI derivatives modified to supress the internal conversion pathway might exhibit more efficient proton transfer on a slower timescale.

As indicated above (and in Fig. 4) the present data suggest that barrierless ESPT occurs to a preformed acceptor site, whilst other solvent-solute orientations do not undergo ESPT (or at least not at a rate that competes with internal conversion). We suggest that the rate of zwitterion formation from the cation is the same as for anion formation because the two reactions follow essentially identical pathways, barrierless proton transfer to a preformed acceptor site.

Finally, it appears that the longest decay time recovered for the directly excited anion is slightly longer than that for the anion formed in the ESPT reaction (1.6 ps compared to 1.2 ps, Table 2). This may simply reflect the limitations of fitting such multicomponent data sets, but we note that the anion formed on ESPT will, on these ultrafast time scales, necessarily occupy a different environment to that for the directly excited anion at higher pH. This arises because immediately following ESPT the positively charged acceptor site will be adjacent to the anion, and may influence its decay kinetics.

We conclude by considering the implications of these data for fluorescent protein photophysics. Firstly, it is apparent that the facile (*ca.* 2 ps) ESPT seen in avGFP requires that the relatively poor photoacid properties of *p*-HBDI are compensated for by a strong proton accepting capability of the proton wire. Further, we speculate that if the stronger photoacid properties of DF*p*-HBDI were transferred to the protein environment, the ESPT reaction would be more thermodynamically favoured than in avGFP. In that case, time resolved measurements would provide important new information on the dynamics of proton transfer along proton wires. For example, one could address the question of whether the rate of ESPT in avGFP is determined by structural dynamics in the multistep proton wire rather than the energetics of the donor and acceptor states. Such experiments are within the range of modern chemical biology, with fluorinated tyrosines having been incorporated into a number of proteins.^32^ Indeed the phenolic ring of EGFP (a pH sensitive mutant of avGFP) has been substituted with single F atoms at both meta and para positions. In this case the effect on the p*K*_a_ of the protein was modest (<0.5 p*K*_a_ units).^33^ The excited state dynamics of such proteins are of considerable interest and will be the subject of further study.

## Conclusions

The photoacid properties of DF*p*-HBDI have been contrasted with those of *p*-HBDI. An exceptionally fast (50 fs) barrierless superphotoacid ESPT reaction has been observed by means of ultrafast time resolved fluorescence upconversion spectroscopy. The ESPT was seen for both neutral and cation forms of the chromophore, and occurs with equal efficiency, the latter giving rise to the rarely observed emission of the zwitterion. The potential for these observations to be exploited in unnatural forms of GFP to probe the dynamics of the proton wire were discussed.

## Supplementary Material

Supplementary information
