# Supplementary material for: Photoacid behaviour in a fluorinated green fluorescent protein chromophore: ultrafast formation of anion and zwitterion states
Source: Chem Sci. 2016 Jun 6;7(9):5747–52. doi: 10.1039/c6sc02031c (PMC5207226; doi:10.1039/c6sc02031c)
Supplement: Supplementary file 1 [file SC-007-C6SC02031C-s001.pdf]

Electronic Supplementary Material (ESI) for Chemical Science.
This journal is © The Royal Society of Chemistry 2016

Photoacid Behaviour in a Fluorinated Green Fluorescent Protein Chromophore: Ultrafast Formation of Anion and Zwitterion States.

Supporting Information

Sergey P. Laptenok,[a] Jamie Conyard,[a] Philip C. Bulman Page,[a] Yohan Chan,[a] Mingxu You,[b] Samie R. Jaffrey[b] and Stephen R. Meech[a]*

[1]School of Chemistry, University of East Anglia, Norwich NR4 7TJ, UK and

[2]Department of Pharmacology, Weill Medical College of Cornell University, 1300 York Avenue, Box 70 New York, NY 10065, USA

* Corresponding author: E-mail: S.Meech@uea.ac.uk

Contents.

1. Description of the fluorescence up-conversion method

2. Figure S1. Absorption and fluorescence spectra of different charge states of p-HBDI in H_2O

3. Figure S2 The fluorescence spectra decomposition

4. Figure S3. Steady state and time resolved emission of the DFp-HBDI in MeOH

5. Figure S4. Fluorescence spectra measured at 400 nm excitation

6. Figure S5. Time resolved emission of DFp-HBDI cation measured at pH 1

7. Table S1. The data recovered from measurements in D_2O.

8. Synthesis and Characterization of DF p-HBDI, Figure S6, NMR Data.

9. Figure S7. Fit data showing the requirement for a risetime in the 500 nnm data (Text Table 2).

Fluorescence up-conversion

Fluorescence up-conversion is a variant of the ultrafast pump-probe methods. In our experiment a Ti:sapphire oscillator produces pulses centred at 800 nm of width 20 fs at a 76 MHz repetition rate and a total power of 800 mW. The laser output was compressed by a prism pair and focussed onto a 50 µm thick Type I BBO crystal to produce up to 15 mW of a 400nm second harmonic pump beam. The gate (800 nm) and pump (400 nm) beams were separated with a dichroic mirror, after which they follow different paths, with a temporal separation controlled by a mechanical delay stage with an accuracy of ca 1 fs. A pair of chirped mirrors was introduced into each beam to compensate for the dispersion introduced by transmission through other optics. The 400 nm pump beam was adjusted to have approx. 8 mW power and was focused with a 150 mm concave mirror to the centre of a 1 mm pathlength cell which contained the sample. The sample was flowed continuously during measurements by a peristaltic pump. The fluorescence emitted from the sample was collected by a reflective microscope objective and focused onto a 100 µm thick Type I BBO crystal where it was mixed with the 800 nm gate beam. A monochromator combined with a photon-counting photomultiplier tube was used to detect the signal generated with a resolution of 15 nm. The intensity of the up-converted signal, at the wavelength selected by the monochromator and the angle of the up-conversion crystal, is proportional to the instantaneous fluorescence intensity during the 20 fs gate pulse, the arrival time of which can be varied by the delay stage. Thus fluorescence up-conversion is an ultrafast sampling technique. The time resolution, measured experimentally by up-converting scattered Raman from the solvent, was determined to be 50 fs and was used in the deconvolution analysis. The apparatus was described in detail elsewhere.[S1]

Figure S1. The pH dependent absorption and fluorescence spectra for HBDI measured in H_2O excited at 380 nm (anion) and 360 nm (neutral and cation). The structures of the neutral (a) cation (b) anion (c) and zwitterion (d) are shown with the most probable charge distribution shown.

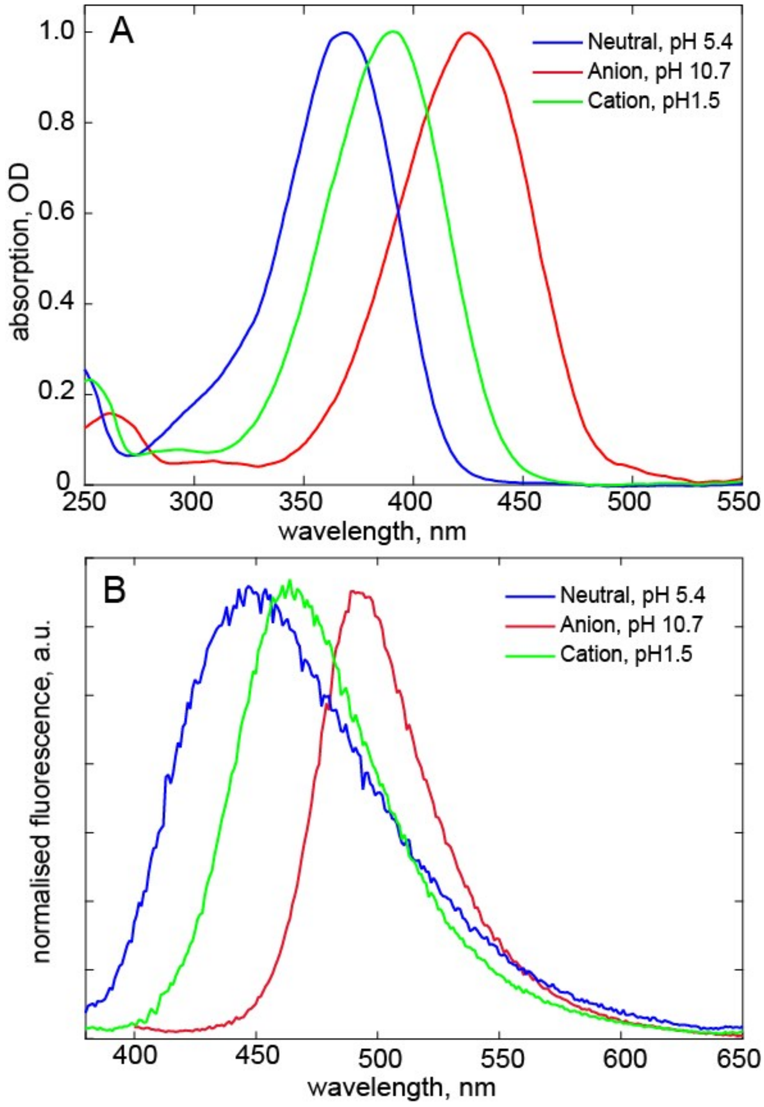

(a)

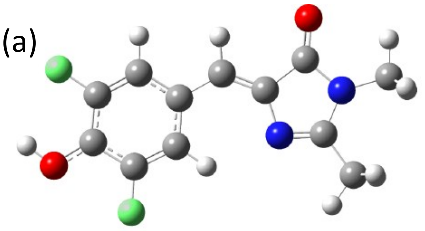

(b)

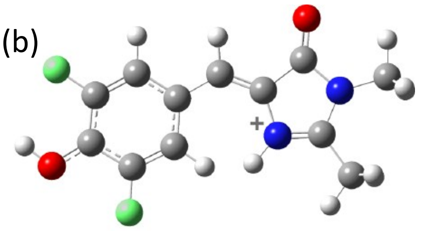

(c)

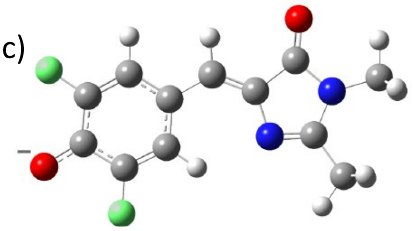

(d)

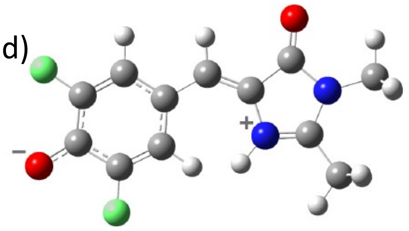

Figure S2. The spectral decomposition (in energy units). The excitation wavelength was 360 nm.

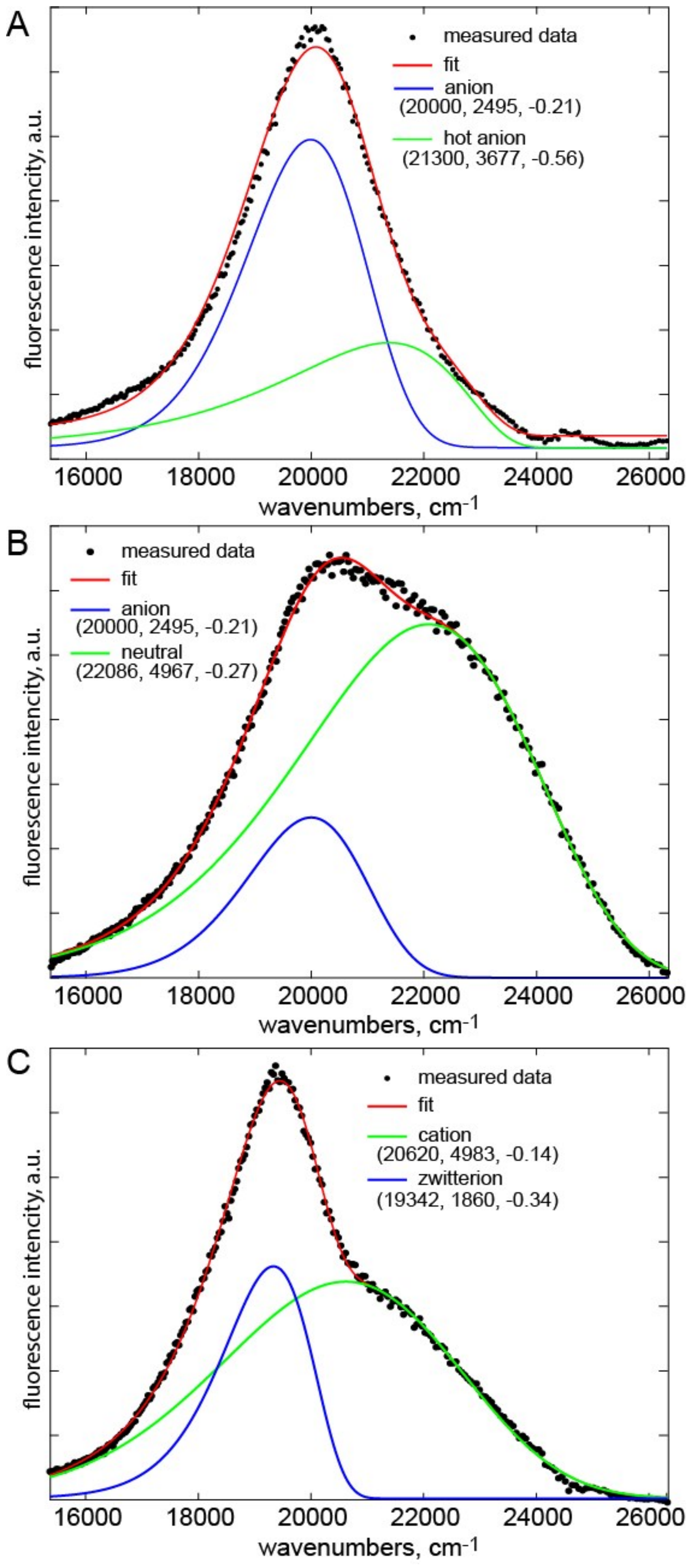

Decomposition of the spectra was achieved by fitting with a sum of two log-normal functions:

$$A(\nu) = A_{max} exp\left(- ln2 \left[\frac{ln\left(1 + 2b\frac{\nu - \nu_{max}}{\Delta\nu}\right)}{b} \right]^2 \right)$$

Where wavenumber $\nu = \lambda^{-1}$, $\Delta\nu$ is related to the full width at half maximum, $A(\nu)$ is a converted fluorescence spectrum $A(\nu) = \lambda^2 * F(\lambda)$ and b is a skewness parameter.[S2]

Fitting was carried out in the energy domain, representation of fit results in wavelengths domain are shown in Figure 2C for ease of comparison; In Figure S2A (Anion) and Figure S2B (Neutral) the shape of anion spectrum was fixed to the one recovered from a single log-normal fit to the anion spectrum excited at 400 nm. The spectrum of the anion excited at 360 nm (A) was found to be significantly broader than that recovered on 400 nm excitation (see below). We ascribe this difference to emission from highly excited vibronic states populated by the more energetic excitation photon; the difference is shown in green. Although such hot band emission is not usually resolved, for very short lived excited states (such as is the case here) it may compete with the relaxed emission. This is consistent with the slightly faster decay observed on the blue edge of the anion emission (Table 2).

Figure S3 Steady state and time resolved emission of the DFp-HBDI in MeOH. Fluorescence measured after excitation using 400 nm.

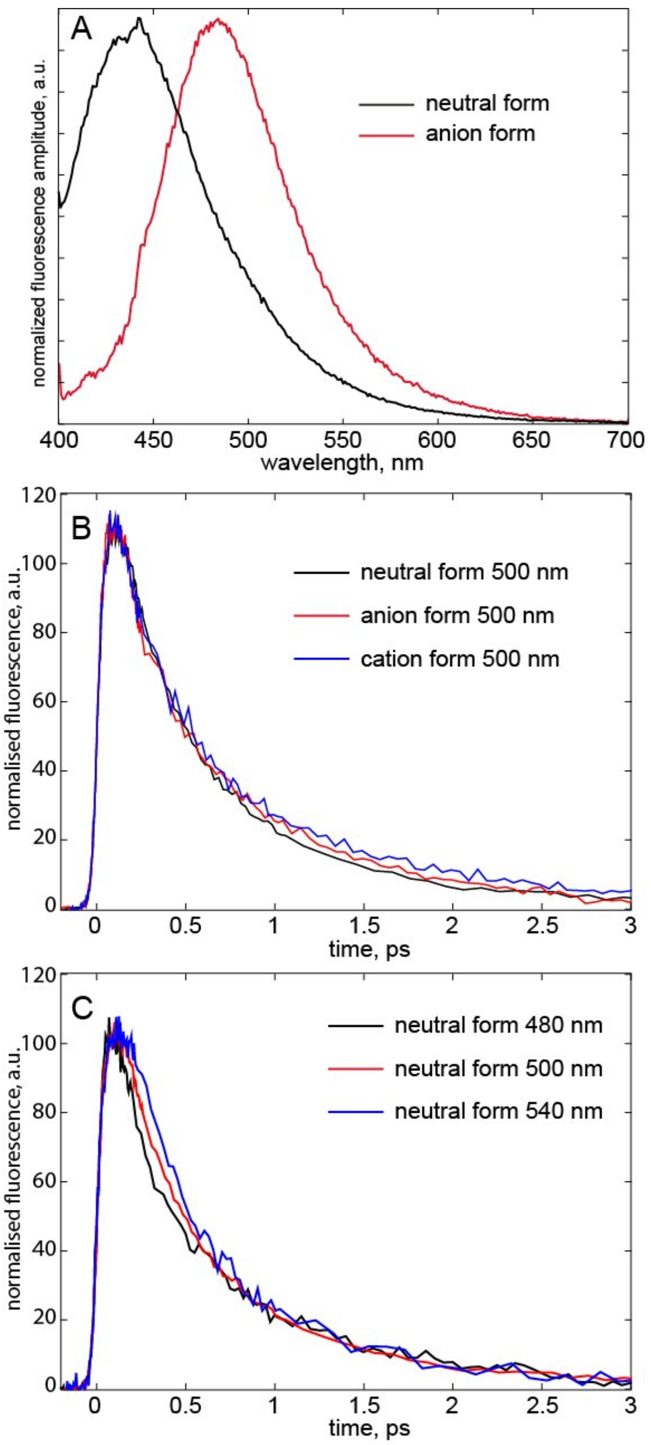

The excited state lifetime decay shows almost no dependence between different species. Fluorescence decay of the species almost mono-exponential without colour dependence. Estimated average lifetimes of species Neutral: 0.56 ps; Anion: 0.62 ps; Cation 0.7 ps.

Figure S4. (A) The pH dependent fluorescence spectra for DFp-HBDI measured at an excitation wavelength of 400 nm. The differences between Figure 2B and Figure S2 are due to the different pH dependent absorbance of the sample (evident in Figure 2A) at the two wavelengths. (B) The spectra could be decomposed into the same components as those at 360 nm, but with different amplitudes; the decomposition of the neutral emission excited at 400 nm is shown for comparison with Figure 2C.

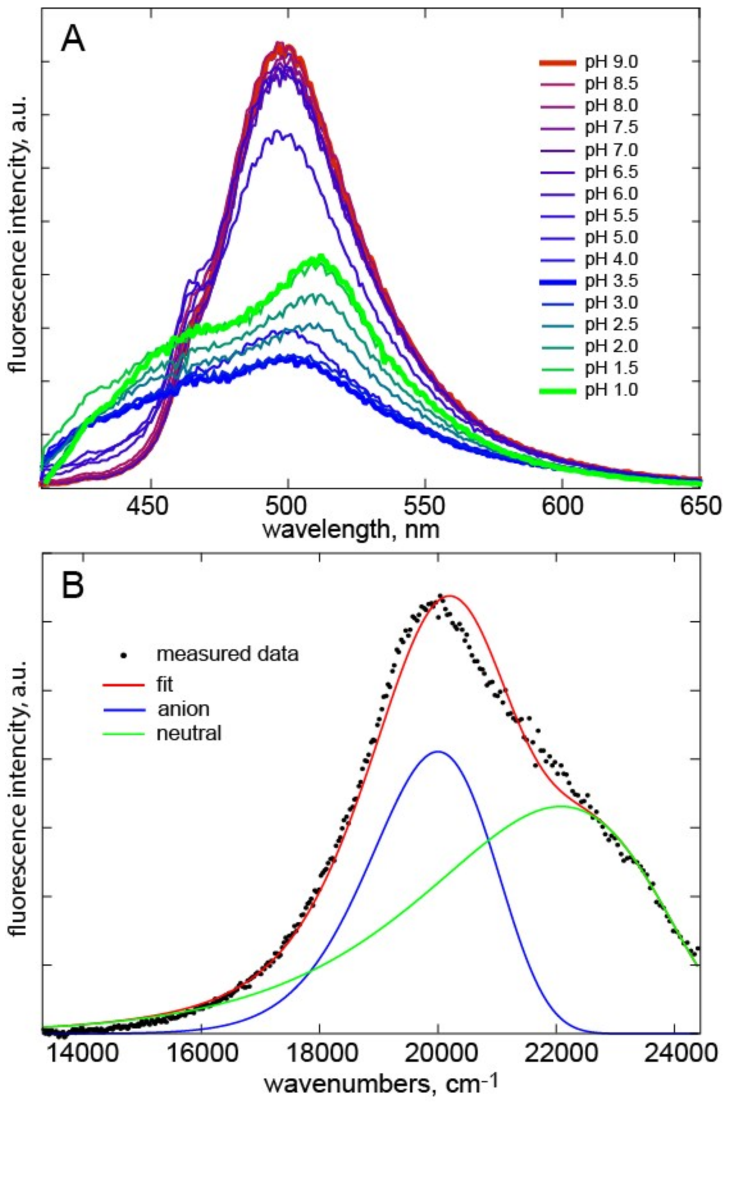

Figure S5 Time resolved emission of the cation excited at 400 nm and measured at emission wavelengths across the spectrum, shown on sub - picosecond (A) and picosecond (B) time scales. Again the need for a risetime in fitting the red-edge data is obvious.

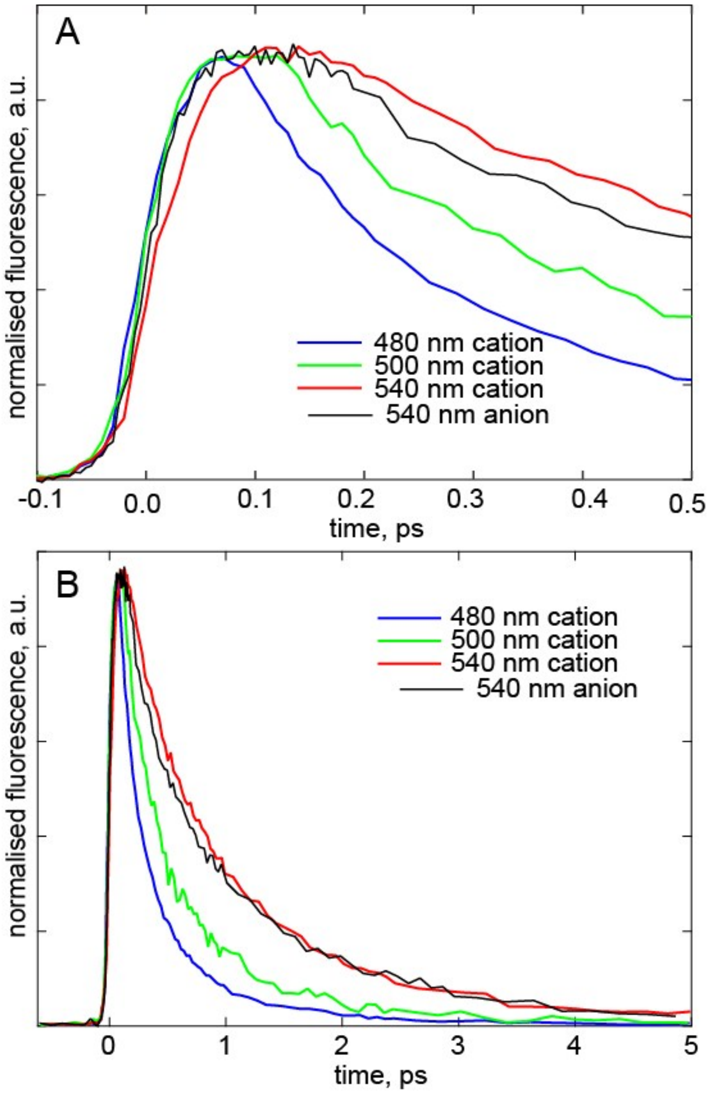

Table S1 Fluorescence decay kinetics for DFp-HBDI measured in D_2O. No isotope effect was observed (Compare with Table 2).

	Detection/ nm	***α***	**τ_1/ ps**	**τ_2/ ps**	**<τ>/ ps**	Rise/ ps
**DFp-HBDI**						
Anion	480	0.60	0.3±0.1	1.4±0.2	0.74	
	500	0.55	0.4±0.2	1.7±0.4	0.98	
	540	0.55	0.4±0.1	1.6±0.4	0.94	
Neutral	480	0.75	0.06±0.1	0.75±0.2	0.23	
	500	0.60	0.25±0.5	1.2±0.3	0.63	
	540	0.47	0.33±0.1	1.3±0.2	0.83	0.06±0.05

Synthesis and Characterization.

DFHBDI was prepared by the method of Jaffrey,[S3] by concomitant cyclization-condensation of 3,5-difluoro-4-acetoxybenzaldehyde with *N*-acetylglycine in the presence of acetic anhydride to give the oxazolone, followed by treatment with methylamine to give the corresponding imidazolone.

3,5-Difluoro-4-acetoxybenzaldehyde

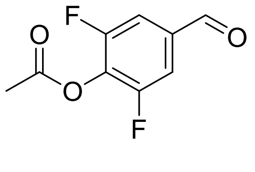

3,5-Difluoro-4-hydroxybenzaldehyde (1.0 g, 6.32 mmol) was dissolved in THF (20 mL), and triethylamine added (1.2 mL, 8.85 mmol, 1.4 equiv.). The solution was cooled to 0 °C and acetyl chloride (0.54 mL, 7.60 mmol, 1.2 equiv.) added slowly. The reaction mixture was stirred overnight at room temperature, filtered through a pad of silica gel, and washed with ethyl acetate. The solvents were evaporated under reduced pressure to yield 3,5-difluoro-4-acetoxybenzaldehyde (1.27 g, 100%).

ν_{max} (film)/cm^{-1}: 2854, 2820, 1791, 1705, 1600, 1510, 1450, 1338, 1178, 1115, 1046, 888, 861, 733. ^{1}H NMR (500 MHz, $CDCl_3$): δ_H 2.42 (3H, s), 7.53 (2H, d, *J*= 7.0 Hz), 9.91 (1H, t, *J*= 2.0 Hz). ^{13}C NMR (125 MHz, $CDCl_3$): δ_C 20.1, 112.9 (d, *J*= 5 Hz), 113.1 (d, *J*= 5 Hz), 132.4, 134.4 (t, *J*= 6 Hz), 155.66 (d, *J*= 250 Hz), 155.70 (d, *J*= 250 Hz), 166.7, 188.6. HRMS (APCI) *m/z* found for $[M+H]^+$: 201.0355; $[C_9H_6F_2O_3+H]^+$ requires 201.0358.

(Z)-4-(3′,5′-difluoro-4′-acetoxybenzylidene)-2-methyl-4,5-dihydro-5-oxo-1,3-oxazole [1]

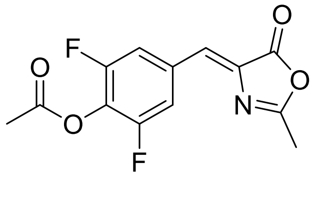

Acetic anhydride (10 mL) was added to a mixture of 3,5-difluoro-4-acetoxybenzaldehyde (1.0 g, 5.00 mmol), *N*-acetylglycine (0.6 g, 5.50 mmol, 1.1 equiv.), and sodium acetate (0.4 g, 5.00 mmol). The mixture was heated under reflux for 4 h, allowed to reach room temperature, and ethanol (10 mL) added. The resulting slurry was cooled overnight in a refrigerator. The suspension was filtered and the collected solid washed with cold ethanol to give the title compound as a yellow solid (0.54 g, 41%).

ν_{max} (film)/cm^{-1}: 1781, 1769, 1666, 1606, 1507, 1434, 1371, 1333, 1186, 1171, 887, 866. ^{1}H NMR (500 MHz, *d6*-DMSO): δ_H 2.42 (3H, s), 2.43 (3H, s), 7.23 (1H, s), 8.09 (2H, d, *J*= 10 Hz). ^{13}C NMR (125 MHz, *d6*-DMSO): δ_C 15.5, 19.8, 115.2–115.8 (m), 125.8, 127.8 (t, *J*=16 Hz), 132.3 (t, *J*= 25 Hz), 134.7, 154.21 (d, *J*= 246 Hz), 155.24 (d, *J*= 246 Hz), 166.8, 167.3, 168.5. HRMS (ESI) *m/z* found for $[M+H]^+$ 282.0575; $[C_{13}H_9F_2NO_4+H]^+$ requires 282.0572.

(Z)-4-(3',5'-Difluoro-4'-hydroxybenzylidene)-1,2-dimethyl-1*H*-imidazol-5(4*H*)-one [1]

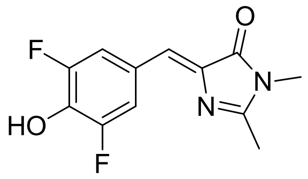

Methylamine (40% in water, 1.7 mL, 10 equiv.) was added to a mixture of (Z)-4-(3',5'-difluoro-4'-acetoxybenzylidene)-2-methyl-4,5-dihydro-5-oxo-1,3-oxazole (0.30 g, 1.07 mmol) and sodium carbonate (0.02 g, 0.16 mmol, 0.15 equiv.). The mixture was stirred at room temperature for 30 min. Methylamine (40% in water, 1.7 mL, 10 equiv.) was added and the mixture heated under reflux for 4 h. The solution was allowed to reach room temperature and water (15 mL) added. The pH of the solution was adjusted to 3 using aqueous HCl (37%). The suspension was cooled overnight in a refrigerator, filtered and the collected solid washed with cold water to give the title compound as a solid (0.21 g, 78%).

ν_{max} (film)/cm^{-1}: 1687, 1651, 1619, 1527, 1443, 1335, 1243, 1136. ^{1}H NMR (500 MHz, *d6*-DMSO): δ_H 2.38 (3H, s), 3.11 (3H, s), 6.92 (1H, s), 7.99 (2H, δ, *J*= 9.0 Hz), 10.93 (1H, s). ^{13}C NMR (125 MHz, *d6*-DMSO): δ_C 15.9, 26.7, 115.6 (δ, *J*= 6 Hz), 115.7 (δ, *J*= 6Hz), 123.2, 125.2 (t, *J*= 9 Hz), 136.1 (t, *J*= 16 Hz), 152.28 (δ, *J*= 240 Hz), 152.34 (δ, *J*= 240 Hz), 138.9, 165.0, 170.1. HRMS (ESI) *m/z* found for $[M+H]^+$ 253.0785; $[C_{12}H_{10}F_2N_2O_2+H]^+$ requires 253.0783.

Figure S6 NMR Data

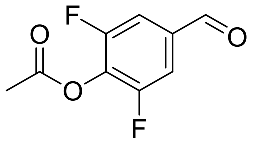
F
O
O
O
F

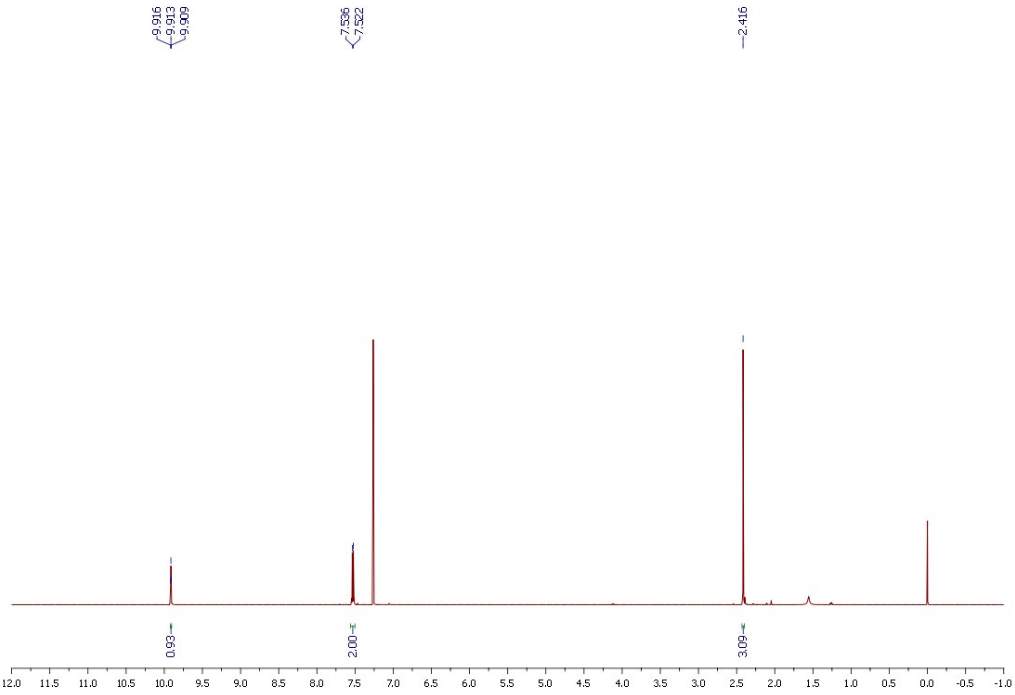
9.916
9.913
9.909
7.536
7.522
2.416
0.93
2.00
3.09
12.0 11.5 11.0 10.5 10.0 9.5 9.0 8.5 8.0 7.5 7.0 6.5 6.0 5.5 5.0 4.5 4.0 3.5 3.0 2.5 2.0 1.5 1.0 0.5 0.0 -0.5 -1.0

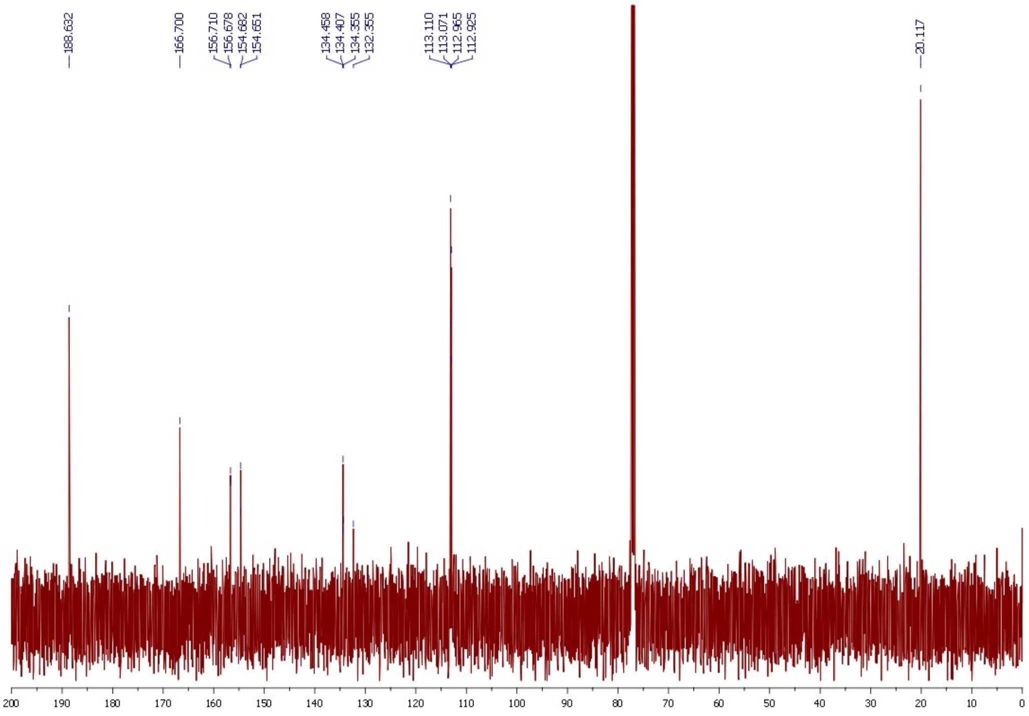
188.632
166.700
156.710
156.678
154.682
154.651
134.458
134.407
134.355
132.355
113.110
113.071
112.965
112.925
20.117
200 190 180 170 160 150 140 130 120 110 100 90 80 70 60 50 40 30 20 10 0

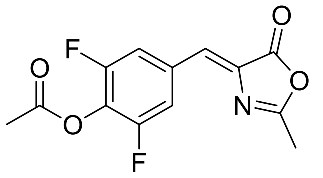
F
O
O
F
O
O
N

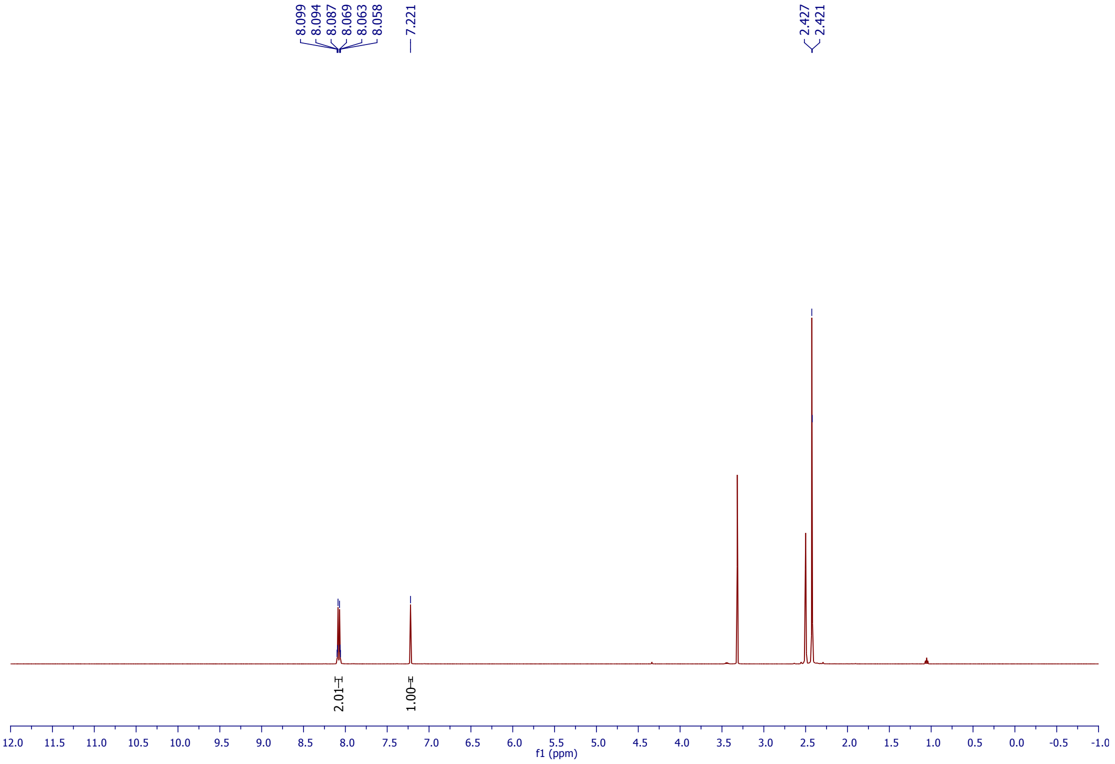
8.099
8.094
8.087
8.069
8.063
8.058
7.221
2.427
2.421
2.01
1.00
12.0 11.5 11.0 10.5 10.0 9.5 9.0 8.5 8.0 7.5 7.0 6.5 6.0 5.5 5.0 4.5 4.0 3.5 3.0 2.5 2.0 1.5 1.0 0.5 0.0 -0.5 -1.0
f1 (ppm)

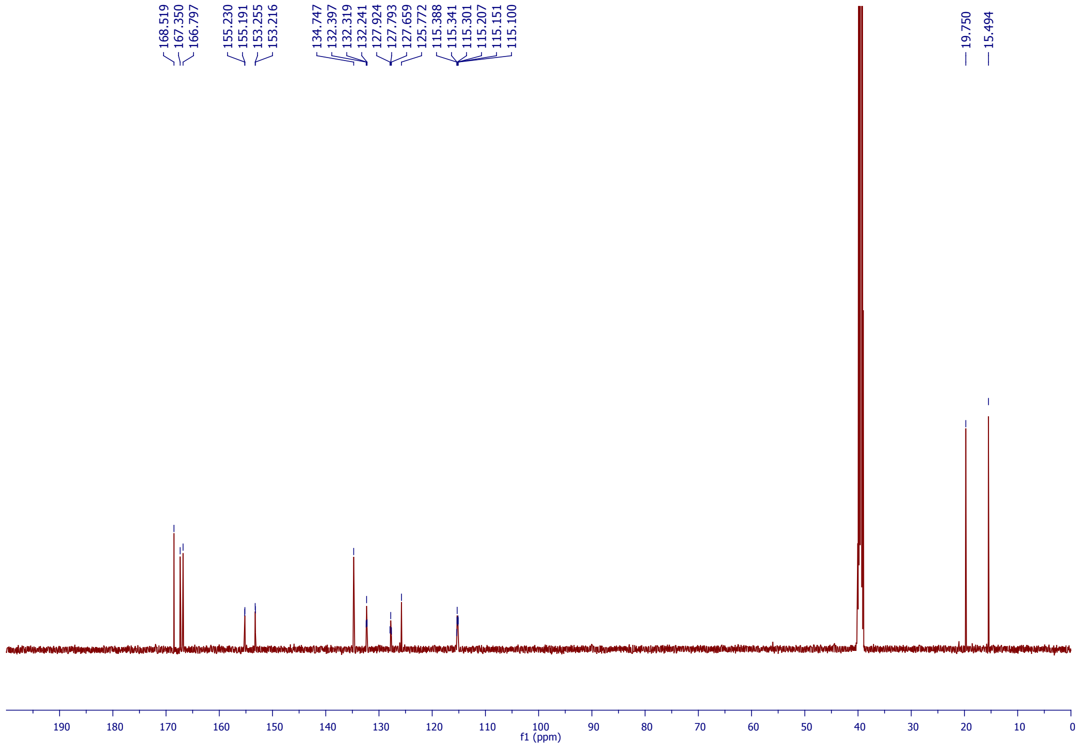
168.519
167.350
166.797
155.230
155.191
153.255
153.216
134.747
132.397
132.319
132.241
127.924
127.793
127.659
125.772
115.388
115.341
115.301
115.207
115.151
115.100
19.750
15.494
190 180 170 160 150 140 130 120 110 100 90 80 70 60 50 40 30 20 10 0
f1 (ppm)

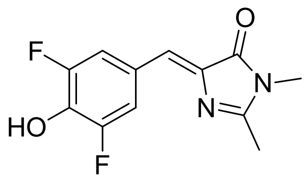

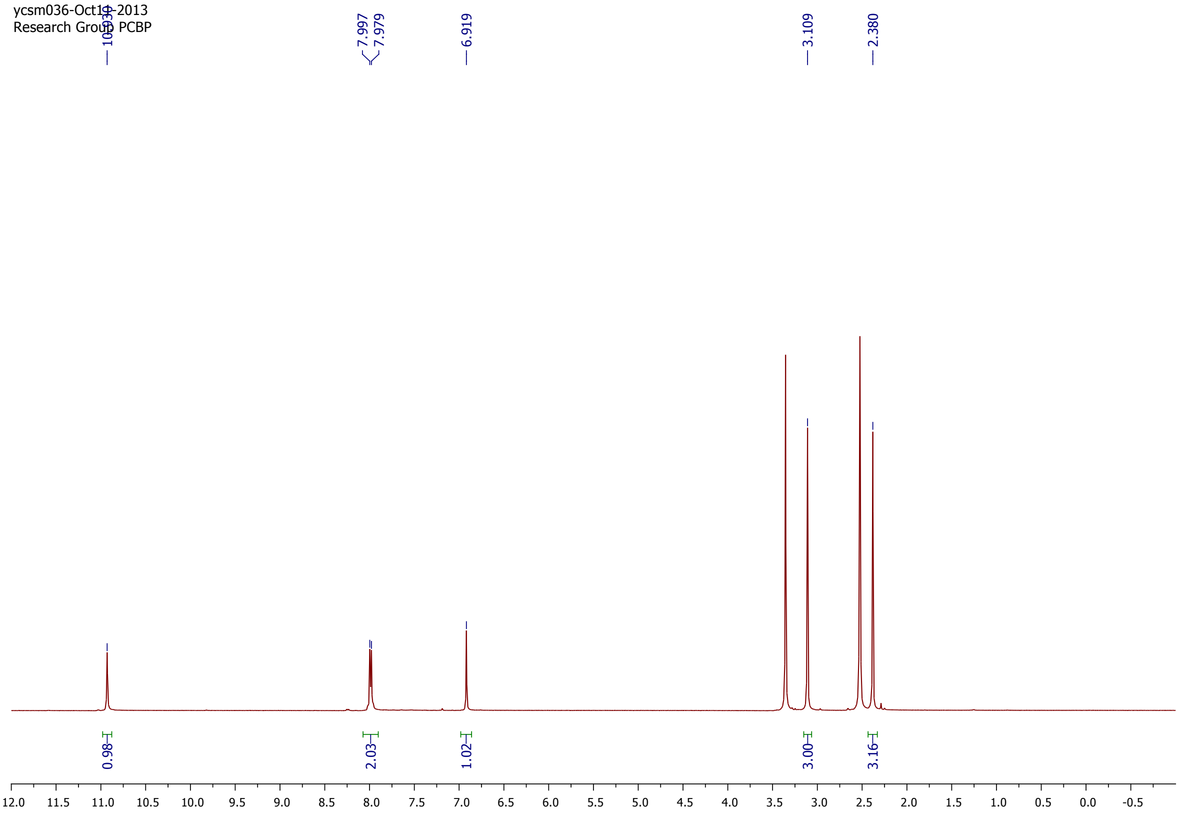
Research Group PCBP
7.997
7.979
6.919
3.109
2.380
0.98
2.03
1.02
3.00
3.16
12.0 11.5 11.0 10.5 10.0 9.5 9.0 8.5 8.0 7.5 7.0 6.5 6.0 5.5 5.0 4.5 4.0 3.5 3.0 2.5 2.0 1.5 1.0 0.5 0.0 -0.5

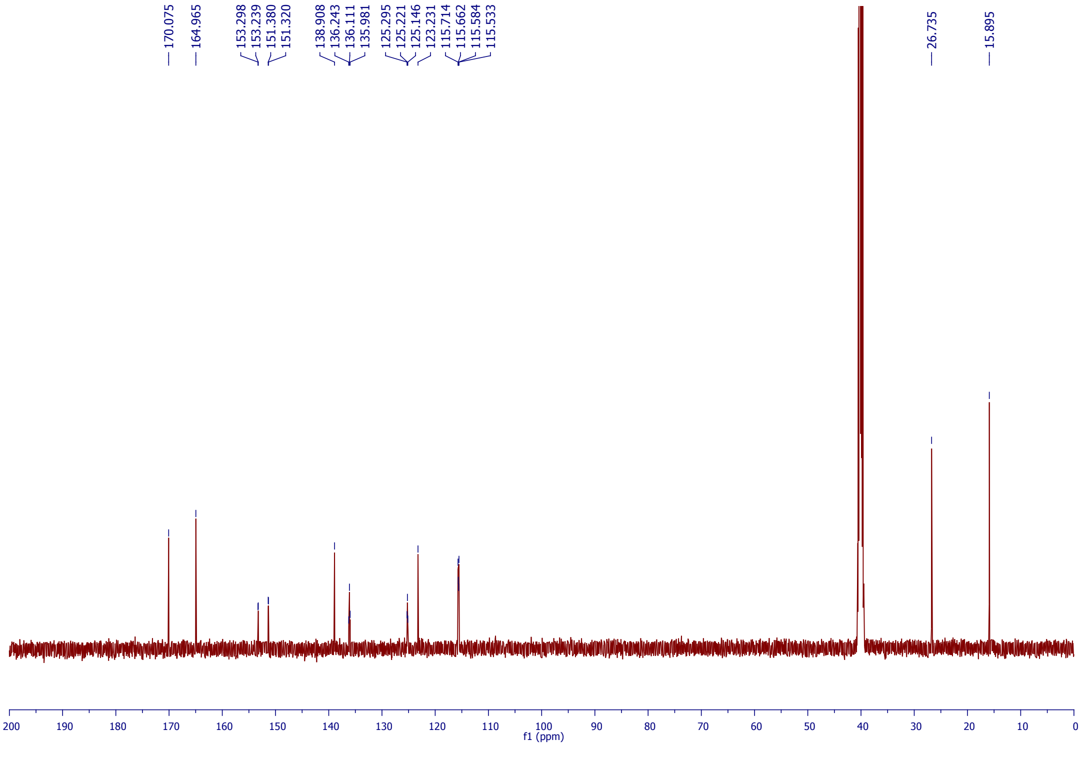
170.075
164.965
153.298
153.239
151.380
151.320
138.908
136.243
136.111
135.981
125.295
125.221
125.146
123.231
115.714
115.662
115.584
115.533
26.735
15.895
200 190 180 170 160 150 140 130 120 110 100 90 80 70 60 50 40 30 20 10 0
f1 (ppm)

Figure S7 Fluorescence up-conversion data for neutral DFp-HBDI measured at 500 nm. The data are fit to just decaying component (red) and rising and decaying components (blue). The residuals reveal the need for the rising component.

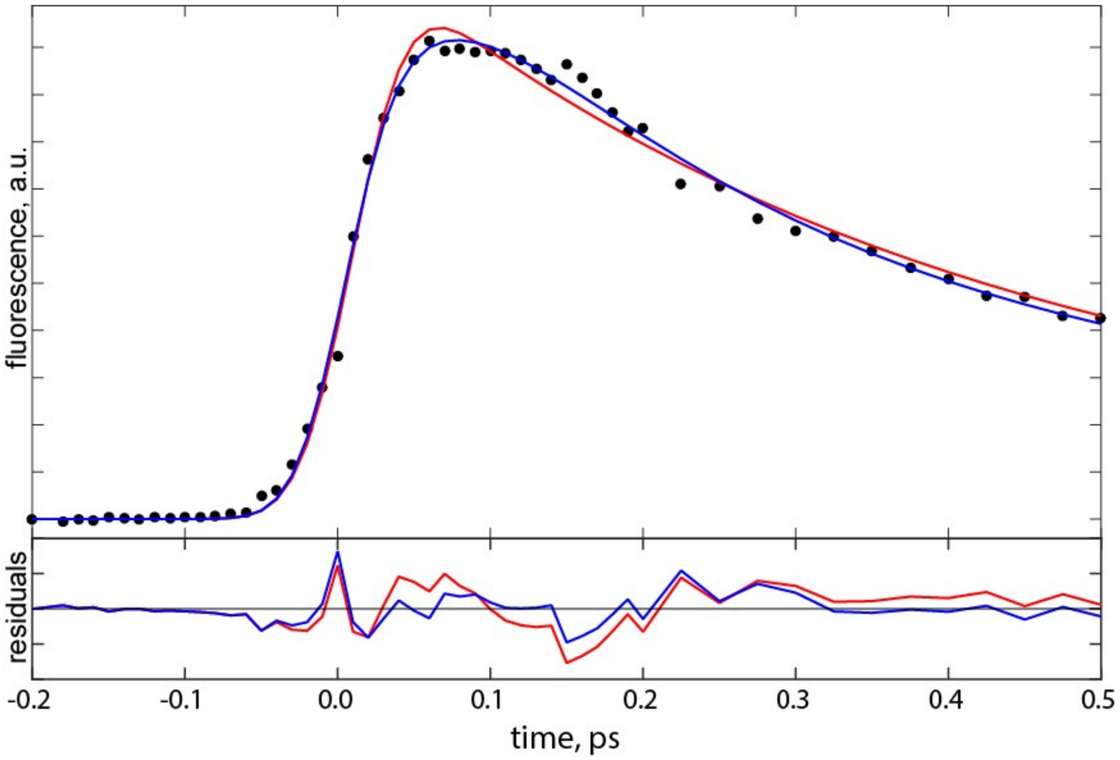

References

S1 Heisler, I. A.; Kondo, M.; Meech, S. R. *J. Phys. Chem*. B **2009**, *113*, 1623

S2 Fraser, R.D. B; Suzuki, E. *Anal. Chem.* **1969**, *41*, 37.

S3 Paige, J. S.; Wu, K. Y.; Jaffrey, S. R. *Science* **2011**, *333*, 642.
